# Editorial: Adaptive Immunity in Pregnancy

**DOI:** 10.3389/fimmu.2021.770242

**Published:** 2021-10-04

**Authors:** Marie-Pierre Piccinni, Sarah Anne Robertson, Shigeru Saito

**Affiliations:** ^1^ Department of Experimental and Clinical Medicine- University of Florence, Florence, Italy; ^2^ Adelaide Medical School and The Robinson Research Institute, University of Adelaide, Adelaide, SA, Australia; ^3^ Department of Obstetrics and Gynecology, University of Toyama, Toyama, Japan

**Keywords:** T helper cells, T reg cells, placenta, spontaneous abortion, pregnancy, maternal-fetal immune tolerance

One of the most remarkable features of reproductive biology is the fact that a healthy woman can successfully carry her genetically disparate conceptus to full term, without immune rejection.

The juxtaposition of the placenta and decidua creates what is referred to as the ‘fetal-maternal interface’, where placental trophoblasts of fetal origin and maternal uterine lymphocytes come into close contact. Due to the presence of paternal class I HLA-C molecules on trophoblasts, the conceptus can be considered to resemble a semi-allograft. Conceptus-derived and placental-derived antigens act to both prime maternal T cells and render the conceptus potentially susceptible to inflammatory effector activity or T cell-mediated attack. After presentation of paternal alloantigens by maternal antigen presenting cells (APCs), the maternal alloantigen-specific T cells proliferate and secrete cytokines, responsible for the activation of allograft rejection or tolerance mechanisms, respectively promoting pregnancy failure or fetal survival.

Therefore, the quality and strength of the adaptive immune response is critical to healthy pregnancy. There is accumulating information that imbalance in the numbers, phenotypes and functional activity of T cell subsets can adversely impact fertility and pregnancy health. Predominant Th1, Th17 and Th17/Th1 immunity and decreased Th2, Th17/Th2 and Treg cells are associated with recurrent pregnancy loss (RPL) of fetuses with normal fetal chromosomal content. Various subsets of T cells are essential for pregnancy tolerance and interact in networks with innate immune cells to counteract inflammation and promote robust placental development. In fact, immune cells that populate the decidua are specialized not only to minimize events that might evoke conceptus attack, but also to foster placental development and function and to combat infections during pregnancy.

In addition, T cells are commonly perturbed in late gestation disorders including preeclampsia, fetal growth restriction and spontaneous preterm birth. There is some evidence that T cell disturbances precede the onset of symptoms and contribute to disease pathophysiology through events around the time of implantation and early placental development.

In this Research Topic we welcomed six original articles and four review articles, which discuss the role of novel immunosuppressive cells and molecules regulating fetal tolerance and development.

During pregnancy, sex steroid hormones like estrogen, progesterone, hCG but also a progesterone-induced mediator, the progesterone-induced blocking factor (PIBF), which conveys some of the immunological effects of progesterone, suppress effector immune activation resulting in successful pregnancy. Csabai et al. reports that the implantation rate is decreased in mice treated with anti-PIBF antibody. In these anti-PIBF-treated mice, NK activity, IL-12A mRNA expression in CD8^+^ T cells and Th1 differentiation are increased, whereas the expression of mRNA for IL-4 (a Th2-type cytokine) is decreased in CD4^+^ T cells (**Figure 1**). Thus, PIBF plays an important role in implantation by upregulating a Th2-type and downregulating Th1-type immune responses.

**Figure 1:**
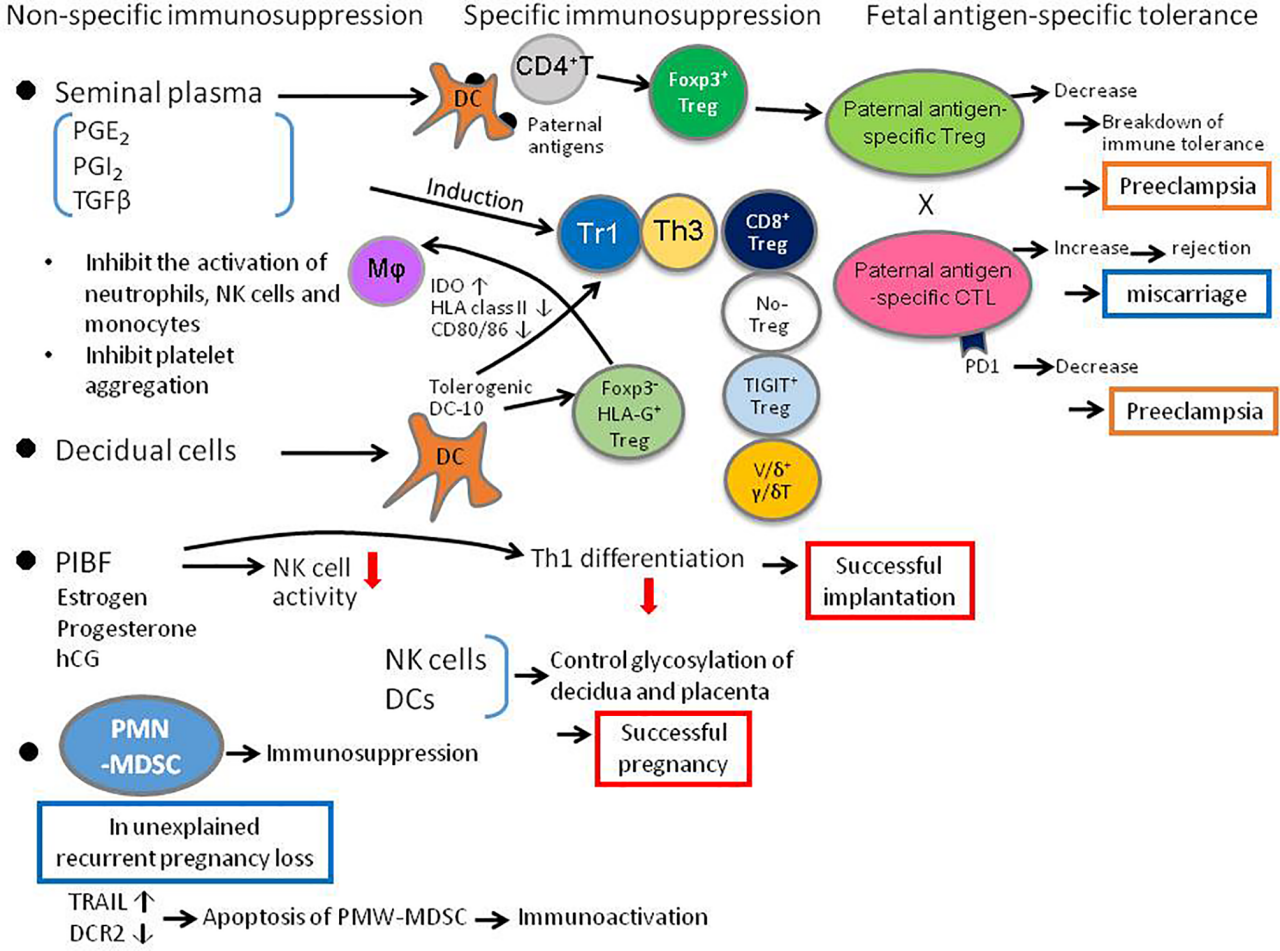
Diagrammatic summary of critical elements of the adaptive immune response to pregnancy, that determine the balance between T cell tolerance and T effector function, and influence the outcome of pregnancy and infant health. DC, dendritic cell; IDO, indoleamine-dioxygenase; MΦ, macrophage, PD1, programmed death 1; PIBF, pregnancy-induced blocking factor; PMN-MDSC, polymorphonuclear myeloid-derived suppressor cell; PGE, prostaglandin E2; PGI, prostaglandin I: Tr1, T regulatory 1; Th, T helper; TIGIT, T cell immunoglobulin and ITIM domain; Treg, regulatory T cell; uNK, uterine natural killer cell.

As well as hormones, other molecules including the prostaglandins (PG) can also regulate immune cells in pregnancy. In particular Andrade et al. show that PGE_2_ also contributes to immune tolerance, by inhibiting platelet aggregation and IL-5 and IL-13 production by innate lymphoid cells (ILCs), and by suppressing neutrophil, NK cell and monocyte effector functions (**Figure 1**).

The role of Treg cells in pregnancy is extensively developed in the Research Topic. Huang et al. reviewed the role of Treg in normal pregnancy, in implantation failure, miscarriage, endometriosis and preeclampsia. Krop et al. described not only the role of well-known Foxp3^+^ Treg cells in pregnancy, and also importantly the role of the lesser-known Foxp3^-^ Treg cells, which include HLA-G Treg cells, Tr1 cells that secrete IL-10 and TGF-β, Th3 cells that secrete TGF-β, IL-10, and IL-4, CD8^+^ Treg cells, NO-Treg cells, TIGIT^+^ Treg cells, and Vδ1^+^γδT cells (**Figure 1**). However, the relationship between these Foxp3^-^ Treg cells and pregnancy disorders remains to be clarified.

Morita et al. examine CD8^+^ T cells that are less well studied in pregnancy, in particular clonally expanded CD8^+^ T cells, using single cell analysis of T cell receptor β (TCRβ) sequences. Clonally expanded CD8^+^ T cells may be a surrogate marker for fetal/paternal antigen-specific CD8^+^ T cells. The authors show that clonal CD8^+^ T cells are more abundant in effector memory CD8^+^ T cells (CD8^+^EM) and that there are more CD8^+^ EM cells in the decidua than in the peripheral blood. The clonal CD8^+^ T cells increase from early to late pregnancy, and PD-1 expression, which suppresses cytotoxic activity, is low on clonal CD8^+^ T cells in early pregnancy but high in late pregnancy. The clonal PD1^-^ CD8^+^ T cells increase in miscarriages with normal fetal karyotype (**Figure 1**). The percentage of clonal CD8^+^ T cells is not different in preeclampsia compared to normal pregnancy, but PD-1 expression is significantly decreased, suggesting an increased cytotoxic activity against fetal antigens in preeclampsia.

Immune cells can not only act by inducing trophoblast tolerance but can also affect the structure of trophoblast by altering the glycan chains of trophoblast. Dendritic cell activity is particularly important, and may be amplified if NK cells are removed as demonstrated by Borowski et al. Thus, immune cells can influence the placental glycade and could impact placental and fetal development (**Figure 1**). The relationship between the alteration of trophoblast glycan chains and immune cells in miscarriage and preeclampsia is an interesting topic for future clarification.

Non-immune cells can also regulate immune cells responsible for immune tolerance in pregnancy. Gori et al. showed that endometrial decidualization increases tolerogenic dendritic cells named DC-10 cells, which secrete IL-10 and induce different regulatory T cells (Treg cells), including HLA-G^+^ Treg cells, Tr1 cells and Th3 cells (**Figure 1**).

Recently, polymorphonuclear myeloid-derived suppressor cells (PMN-MDSCs) have been found to increase in the pregnant uterus and play an important role in maintaining pregnancy. Li et al. show that the number of decidual PMN-MDSCs decrease in patients with unexplained recurrent pregnancy loss. PMN-MDSC apoptosis, increased by elevated TRAIL and decreased by DcR2, could explain the decreased number of PMN-MDSCs in unexplained recurrent pregnancy loss (**Figure 1**). The interaction between PMN-MDSCs and immune cells such as Treg cells, need to be investigated.

Van der Zwan et al. used mass cytometry to analyze lymphocyte subpopulations in the decidua and peripheral blood. Such analysis could be helpful for the classification of immunocompetent cells and to clarify the role of each of these cells in the decidua. This promising technology, which may serve as a foundation for further identification of immune subsets in healthy and complicated pregnancy, is set to offer further advances in the future.

Finally, it is important to recognize that the maternal adaptive immune response to pregnancy has consequences not only for pregnancy outcome, but also for the health of the child after birth. Albrecht et al. review emerging studies showing that cellular immunity is transferred from mother to child not only through IgG transfer during pregnancy, but also by maternal cellular immunity transmitted to the child through lactation after birth. This article introduces the interesting possibility of transferring immunity to the fetus by vaccination during pregnancy.

In summary, this collection of papers provide a snapshot of the state of this field and provide new insight on the mechanisms and significance of the adaptive immune response to maternal and infant health. Collectively the work highlights the imperative to further delineate the underlying mechanisms by which maternal tolerance is generated and mediated, so that interventions to protect against immune-based pregnancy disorders arising from compromise maternal tolerance can be advanced.

## Author Contributions

All authors listed have made a substantial, direct, and intellectual contribution to the work and approved it for publication.

## Conflict of Interest

The authors declare that the research was conducted in the absence of any commercial or financial relationships that could be construed as a potential conflict of interest.

## Publisher’s Note

All claims expressed in this article are solely those of the authors and do not necessarily represent those of their affiliated organizations, or those of the publisher, the editors and the reviewers. Any product that may be evaluated in this article, or claim that may be made by its manufacturer, is not guaranteed or endorsed by the publisher.

